# Supplementary material for: Role of the Hippocampus During Logical Reasoning and Belief Bias in Aging
Source: Front Aging Neurosci. 2020 May 5;12:111. doi: 10.3389/fnagi.2020.00111 (PMC7232576; doi:10.3389/fnagi.2020.00111)
Supplement: Supplementary file 2 [file Presentation_1.pdf]

Supplementary Results

Behavioral results

Reaction Times

Repeated measures ANOVA on reaction times showed a significant main effect of conclusion (*F*(1,57) = 15.73, *p* = .000, η_p^2 = .21), suggesting that participants responded faster to unbelievable conclusions (*M* =5.60, *SD* =1.49) relative to believable ones (*M* =5.86, *SD* =1.53). The main effect of group was significant (*F*(1,57) = 31.83, *p* = .000, η_p^2 = .35). Older adults responded slower than younger adults. Furthermore, the interaction between conclusion and premise was significant (*F*(2,114) = 5.21, *p* = .007, η_p^2 = .08), suggesting that when premises were believable or neutral, participants were faster to respond to unbelievable conclusions relative to believable ones (believable: *t*(58) = 3.69, *p* = 0.000, *d* = .96; Neutral: *t*(58) = 3.97, *p* = 0.000, *d* = 1.04). No difference was found for unbelievable premises and all other effects were not significant (*Ps* > .05).

Rejection rate

Repeated measure ANOVA on rejection rates revealed a significant main effect of premise belief load (*F*(2,114) = 10.11, *p* = .000, η_p^2 = .15), suggesting that believable premises had lower rejection rates relative to unbelievable and neutral ones (*t*(58) = 2.95, *p* = 0.004, *d* = .77 and *t*(58) = 2.32, *p* = 0.02, *d* = .60, respectively). A significant main effect of conclusion belief load (*F*(1,57) = 10.30, *p* = .002, η_p^2 = .15) suggested that believable conclusions had a lower rejection rate relative to unbelievable conclusions (*t*(58) = 3.07, *p* = 0.003, *d* = .80). Also, the interaction between conclusion and premise was significant (*F*(2,114) = 8.39, *p* = .000, η_p^2 = .12). Unbelievable premises (*M* =.92, *SD* =.22) had a higher rejection rate than believable premises when the conclusion was believable (*M* =.73, *SD* =.27; *t*(58) = 6.08, *p* = .000, d = 1.59). A significant interaction between age group and conclusion (*F*(1,57) = 8.61, *p* = .005, η_p^2 = .13) suggested that older adults had a higher rejection rate for unbelievable conclusions (*M* =.88, *SD* =.16) than for believable ones (*M* =.74, *SD* =.23; *t*(29) = 3.61, *p* = .001, *d* = 1.34). This was not observed in younger adults (*t*(28) = .31, *p* = .75, *d* = .11). No other effects were significant (all *p*s > .05).
