# Supplementary material for: Role of the Hippocampus During Logical Reasoning and Belief Bias in Aging
Source: Front Aging Neurosci. 2020 May 5;12:111. doi: 10.3389/fnagi.2020.00111 (PMC7232576; doi:10.3389/fnagi.2020.00111)
Supplement: Supplementary file 3 [file Image_1.pdf]

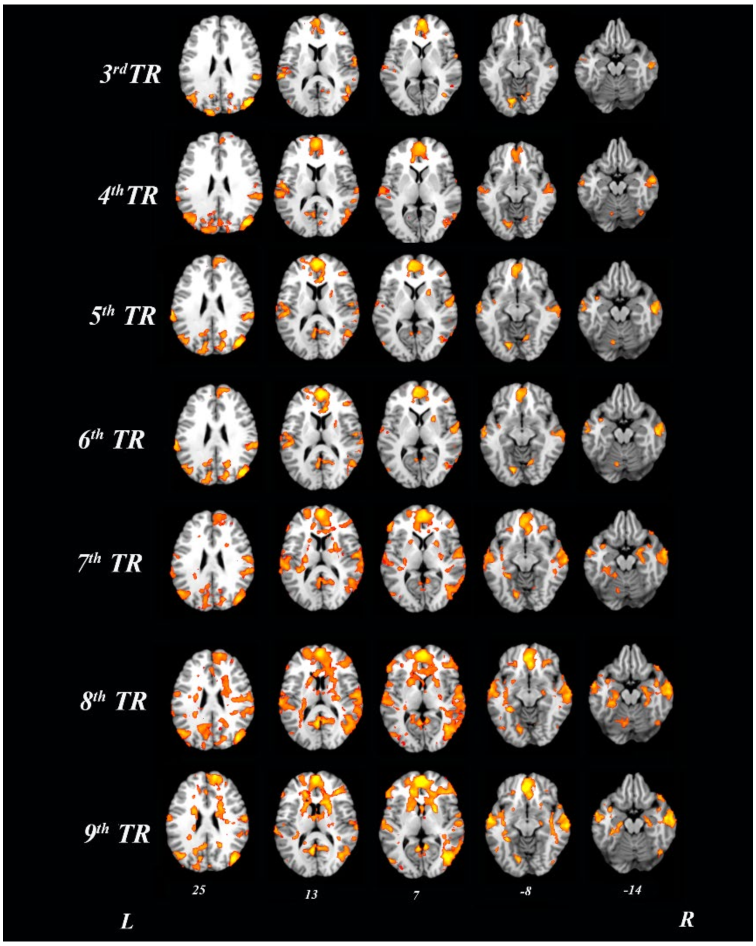

Supplementary Figure 1. Whole-brain task-related results during decision-making. Onsets of the conclusion stage for all six experimental conditions were used for this analysis and results reported in multiple TRs, from 3rd TR (around 1.96 sec) to 9th TR (around 5.89 sec). Older adults recruited these areas more than their younger counterparts for all conditions. All reported regions have bootstrap ratio ≥ 3.5 and cluster size ≥ 50 voxels. Abbreviations: L = left hemisphere, R = right hemisphere.
